# The geographical and seasonal effects on the composition of marine microplastic and its microbial communities: The case study of Israel and Portugal

**DOI:** 10.3389/fmicb.2023.1089926

**Published:** 2023-02-22

**Authors:** Katherine S. Marsay, Ana C. Ambrosino, Yuri Koucherov, Keren Davidov, Neusa Figueiredo, Iryna Yakovenko, Sheli Itzahri, Marta Martins, Paula Sobral, Matan Oren

**Affiliations:** ^1^Department of Molecular Biology, Ariel University, Ariel, Israel; ^2^MARE - Marine and Environmental Sciences Centre & ARNET - Aquatic Research Network Associated Laboratory, NOVA School of Science and Technology, NOVA University of Lisbon, Lisbon, Portugal; ^3^DCEA - Department of Environmental Sciences and Engineering, NOVA School of Science and Technology, NOVA University of Lisbon, Lisbon, Portugal

**Keywords:** 16S, marine bacteria, metabarcoding, nanopore MinION, microplastics, biodiversity, plastic polymers, plastisphere

## Abstract

**Introduction:**

Floating microplastic debris are found in most marine environments around the world. Due to their low density and high durability, plastic polymers such as polyethylene, polypropylene, and polystyrene serve as stable floating substrates for the colonization of diverse communities of marine organisms. Despite the high abundance of microplastic debris in the oceans, it is not clear how the geographical location and season affect the composition of marine microplastic and its bacterial microbiome in the natural environment.

**Methods:**

To address this question, microplastic debris were collected from the sea surface near estuaries in the Mediterranean Sea (Israel) and in the Atlantic Ocean (Portugal) during summer and winter of 2021. The microplastic physical characteristics, including shape, color, and polymer composition, were analyzed and the taxonomic structure of the microplastic bacterial microbiome was characterized using a high-resolution metabarcoding pipeline.

**Results:**

Our results, supported by previously published data, suggest that the plastisphere is a highly diverse ecosystem which is strongly shaped by spatial and temporal environmental factors. The geographical location had the highest impact on the plastisphere physical characteristics and its microbiome composition, followed by the season. Our metabarcoding analysis showed great variability between the different marine environments with a very limited microbiome “core.”

**Discussion:**

This notion further emphasizes the importance of plastisphere studies in different geographical locations and/or seasons for the characterization of the plastisphere and the identification of plastic-associated species.

## Introduction

Between 13 and 23 million tons of plastic enter the oceans every year and with this figure expected to rise, marine plastic pollution is an increasing environmental concern (Borrelle et al., 2020; Lincoln et al., 2022). Most plastic pollutants enter the marine environment from coastal and inland sources by wind and water, either directly or through treated/untreated waste water (Uddin et al., 2020). A major channel for land plastic waste arrival to the sea are rivers and estuaries (Mai et al., 2020; Yang et al., 2021). After arrival to the ocean, plastic pollutants circulate the marine environment for many years during which they are slowly fragmented into smaller microplastic (<5 mm) and nanoplastic (<1 μm) particles. The fragmentation is driven by thermal- and photo-degradation, abiotic/biotic oxidation or hydrolysis (Andrady, 2011) along with the sheering forces of currents and waves.

High microplastic concentrations were recorded in five subtropical ocean gyres (Cózar et al., 2017); however, microplastic debris has been found in almost all marine environments including the Arctic and the Antarctic regions (Peeken et al., 2018; Lacerda et al., 2019; Cunningham et al., 2020). Enclosed basins that are surrounded by densely populated coasts such as the Mediterranean Sea serve as hubs for microplastic pollution (Sharma et al., 2021). Marine microplastic consist of debris that are made of a variety of different polymers, additives, and dyes. They include primary microplastics that are manufactured in microscopic sizes or secondary microplastics that are derived from larger plastic objects (Cole et al., 2011). Polymers with lower density than sea water, including polyethylene (PE), polypropylene (PP), and polystyrene (PS), dominate the sea surface, whereas denser polymers, such as polyethylene terephthalate (PET) are enriched in deeper water and on the sea floor (Erni-Cassola et al., 2019).

At sea, microplastics are readily colonized by a variety of marine organisms, creating an ecosystem that is distinct from its surrounding water which is referred to as the plastisphere (Zettler et al., 2013). It was shown that the plastic microbiome composition is variable among different geographical locations (Oberbeckmann et al., 2014; Amaral-Zettler et al., 2015; Coons et al., 2021) and that it is affected by environmental factors such as salinity, water temperature, light, and nutrient availability (Amaral-Zettler et al., 2015; Oberbeckmann et al., 2018; Misic and Covazzi Harriague, 2019). Although the colonization characteristics of microbial communities on microplastics have been investigated in a variety of short-term (Oberbeckmann et al., 2014, 2016; Coons et al., 2021; Zhang et al., 2022) and long-term (Pinnell and Turner, 2020) *in-situ* experiments, only few studies have investigated the effects of both geographical and seasonal factors in the natural environment (Dong et al., 2021; Yang et al., 2021).

To understand how seasonal and geographical factors may affect the plastisphere composition, we sampled microplastic debris in two separate marine environments, near river estuaries in the Mediterranean Sea (Israel) and in the Atlantic Ocean (Portugal), in two opposing seasons–summer and winter. The physical composition of the microplastic debris as well as the taxonomic structure of the bacterial communities attached to them was obtained, analyzed, and compared.

## Methods

### Sampling

**Figure 1:**
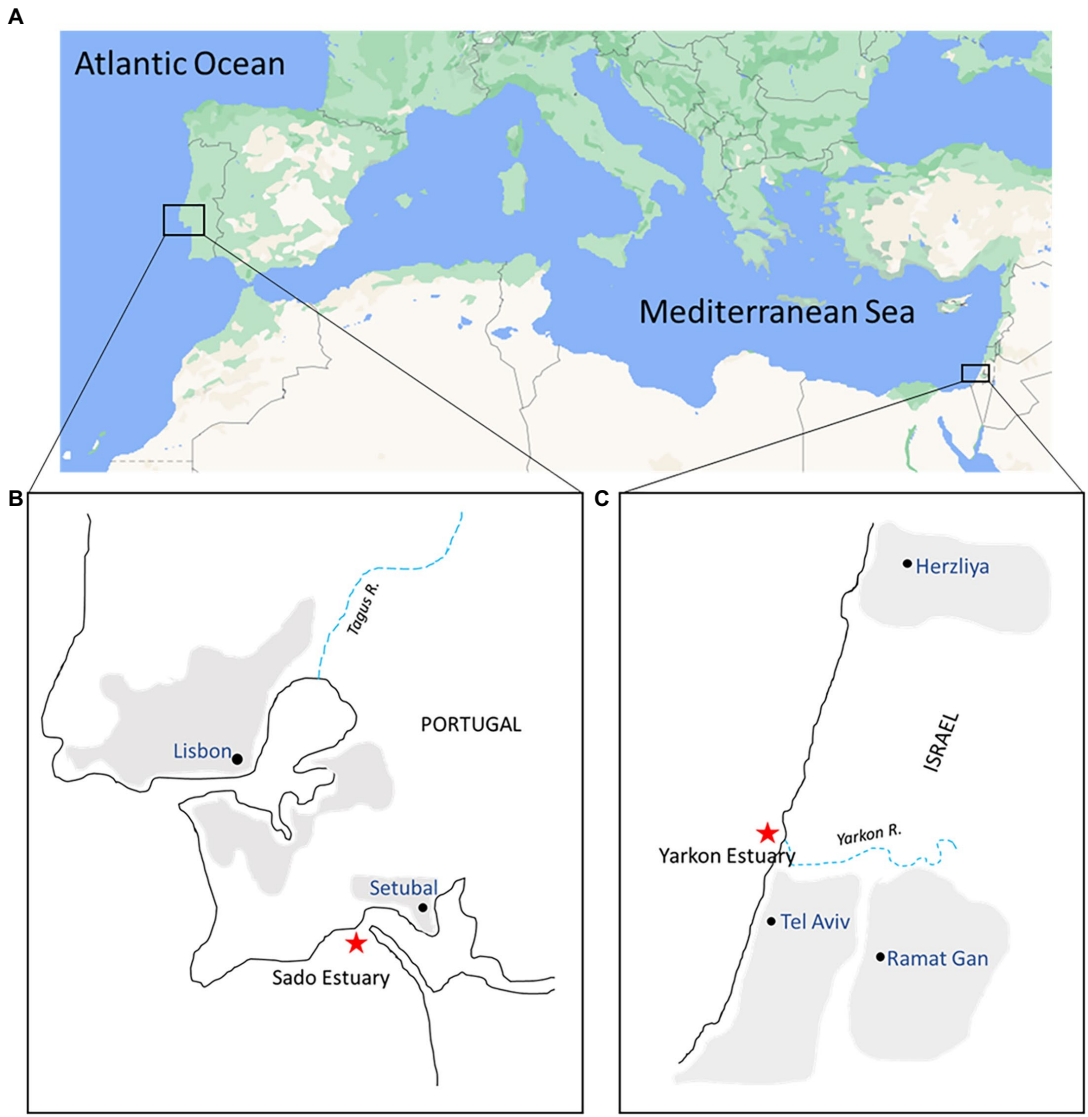
Map of collection sites. **(A)** Sampling areas in the eastern Atlantic Ocean (Portugal) and the eastern Mediterranean Sea (Israel); **(B,C)** Enlargement of the insets from **(A)**. Red stars indicate the Portugal and Israel sampling sites near the mouth of both estuaries.

The microplastic and seawater were sampled from two sites representing two separate marine environments–one in the Atlantic Ocean (Portugal site) and one in the eastern Mediterranean Sea (Israel site). The exact locations were 0.5–1.2 km from the shoreline of Yarkon estuary, Tel Aviv, Israel (32°06′35.7”N, 34°46′18.7″E) and of the Sado estuary, Setubal, Portugal (38°28′22.2”N 8°58′28.3”W; Figure 1). The samples were collected from the sea surface in both locations in the winter (February/March) and in the summer (July) of 2021 using a manta net (Hydro-Bios, Microplastic net, 438,217), with a mesh size of 300 μm and mouth opening of 30 × 15 cm. The manta net was towed from a research vessel at a speed of 2–3 knots for 20–30 min in parallel to the coastline (3 times on the same line). The water volume filtered through the net during each tow was calculated from flow meter counts (Hydro-Bios-438,110) using the expression number of revolutions x 0.3 x net opening area (m^2^)/1000 = volume m^3^ (Supplementary Table S1). Physico-chemical parameters (water temperature, salinity, and depth) were registered (Table 1) with a portable multiparameter probe (HI98194 Multiparameter, HANNA Instruments).

**Table 1:** Environmental parameters and microplastic concentration for each collection.

	Yarkon summer	Yarkon winter	Sado summer	Sado winter
Date	13/07/2021	03/02/2021	21/07/2021	02/03/2021
Sea temperature (°C)	29.8	19.6	18.0	15.3
Salinity (PSU)	39.46	39.47	37	34.3
Site depth (m)	6.3	6.2	5.6	6.2
Microplastic concentration (particles m^−3^, mean ± SD)	112.59 ± 13.18	0.07 ± 0.03	0.04 ± 0.02	0.08 ± 0.04

Microplastic particles recovered from the collecting cod-end of the net were rinsed in filtered artificial seawater. Seawater from the site was sampled using sterile sampling bottles. For DNA metabarcoding analysis of the plastic microbiome, 10 microplastic particles were randomly picked up from each sample and the rest was kept in sea water for the characterization of the microplastic. For DNA metabarcoding analysis of the water microbiome, 0.5 L of the sampled water was filtered onto a 0.22 μm polyethersulfone membrane (Millipore) using a 20 l/min pump (MRC).

### Identification and quantification of microplastics

The physical analysis of all microplastic samples was performed in Portugal. Samples were first processed to remove organic material. A chemical digestion using potassium hydroxide (KOH) was used to extract the microplastics from biological matrices, according to (Pagter et al., 2018; Supplementary information, protocol, SI-P1) and analyzed in entirety except for the Yarkon samples from summer which due to the high amount of microplastics present were subsampled six times using a Folsom Plankton Splitter (Wildco® 1831-F10). All plastic particles were observed using a stereoscopic microscope (Leica DFC480) equipped with a camera (Leica Flexacam). The particles were then counted, measured, and separated according to their typology (fragment, filament, pellet, film, microbead, and sponge/foam) and color (black, blue, white, transparent, red, green, multicolor, and others). Examples for typologies and colors are presented in Supplementary Figure S1.

ImageJ software (v1.48, National Institutes of Health) and Leica LAS-X measurement software (Leica Application Suite X-LAS X) were used to measure the microplastics. The plastic particles that were removed for DNA extraction were also included in the total plastic particles. A blank was used (filter exposed to air) throughout the procedure and microplastics found in the blanks that were similar to those found in the samples were removed from the analyzes.

IBM® SPSS® Statistics 25.0 software was used for the statistical analysis of the microplastics. Kolmogorov–Smirnov and Shapiro–Wilk tests were used to test the normality of variables. Nonparametric tests were selected since the data did not follow a normal distribution. To compare the abundance of plastics, between seasons (summer and winter) and locations (Yarkon and Sado) the non-parametric Kruskal-Wallis H (KW) test was used. Mann Whitney U-test was used to perform Pairwise comparisons between two independent groups. A significance level of 0.05 was considered for all statistical analyzes.

Fourier transformed infrared spectroscopy (FTIR Spectrum Two™ ATR Universal spectrometer, Perkin Elmer, United States) in attenuated total reflectance (ATR) was used to collect spectra in the absorbance mode in the region of 4,000 cm-1 to 450 cm-1 with a data range of cm-1. The resolution was fixed at 4 cm-1 (4 scans). Plastic samples were pressed against the diamond crystal with a force of 80–120 N. Spectra were obtained by absorbance (A) and analyzed using PerkinElmer Spectrum IR software (version 10.7.2). The results were compared with several reference spectra from different Perkin Elmer databases and pre-existing spectral libraries (Hummel, 2002; Coates, 2006; Jung et al., 2018) to identify the chemical nature of the plastics. The acceptance level was established at >90% similarity match, between the sample spectrum and the reference spectra database. Statistical analyzes were performed using IBM® SPSS® Statistics 25.0 software (Chicago, IL, United States).

### PCR and MinION sequencing

DNA was extracted from all samples using DNeasy PowerWater Kit (Qiagen) according to manufacturer instruction. Water DNA was extracted directly from the polyethersulfone filters, whereas plastic biofilm DNA was extracted directly from the washed microplastic debris (10 particles in each pooled sample). The amplification, sequencing, and bioinformatics were performed in-house in Israel according to our previously tested pipeline (Davidov et al., 2020). The complete 16S rRNA gene was amplified using 27F (5′- AGAGTTTGATCMTGGCTCAG-3′) and 1492R (5′- GGTTACCTTGTTACGACTT-3′) primers (Weisburg et al., 1991), with an expected product size of ~1.5 kb. The PCR amplification parameters were: initial denaturation at 94°C for 2 min, followed by 32 cycles of denaturation at 94°C for 30 s, annealing at 56°C for 30 s, extension at 72°C for 45 s, and single extension at 72°C for 5 min. The reaction volume was 50 μl with 25–75 ng of template sample DNA. The PCR products were cleaned with QIAquick-PCR Purification kit (QIAGEN) to meet the criteria of the MinION nanopore library preparation protocol (Karamitros and Magiorkinis, 2018). The sequencing library was multiplexed using the 16S barcoding kit 1–24 protocol with SQK-16S024 kit (Oxford Nanopore Technologies), loaded onto to the MinION Nanopore Spot-on flow cell (FLO-MIN106D, version R9) and sequenced until reaching ~7 Giga nucleotides (~4 M reads). Base-calling for all libraries was done by the Guppy base calling software 3.3.3, using the MinKnow program with the “high accuracy” option. Raw reads were obtained in FAST5 and FASTq formats from which “pass” quality reads were subjected to further analysis. All Nanopore MinION filtered reads analyzed in this project were deposited in the NCBI SRA database^1^ under BioProject PRJNA795711 (accession numbers: SRX13680174-97).

### Sequence analysis and bioinformatics

Processing and analysis of reads was performed using MetONTIIME pipeline and QIIME2 plugins (Bolyen et al., 2019). Reads were demultiplexed and adaptors and PCR primers were trimmed. Sequences were filtered based on nanopore read quality (min_quality; 8), and read length was restricted (amplicon_length X, lenfil_tol Y) based on read length histograms, to give the range of 1,200–1,600 nucleotides. Clustering and taxonomic classification was performed separately for each barcode. Sequences were clustered into consensus sequences with default MetONTIIME pipeline parameters (*de novo* strategy, clustering threshold parameter perc-identity 1). Consensus sequences of the 16S rRNA genes were compared to the Silva132 database (Quast et al., 2013) using BLAST with 80% similarity threshold to generate operational taxonomic units (OTUs). The featured tables were imported into R using phyloseq (R Core Team, 2018). Alpha diversity was estimated by richness (observed Operational Taxonomic Units (OTUs)) and with Shannon diversity indexes using phyloseq (McMurdie and Holmes, 2013). After alpha diversity analysis, OTUs with a total abundance of 1 were excluded and the remaining OTUs were normalized to relative read abundance (dividing the number of reads for each sample by the total reads count). All further analyzes were based on relative read abundance. Phyloseq (McMurdie and Holmes, 2013) was used to perform principal coordinates analysis (PCoA) with Bray–Curtis distance matrix to visualize multivariate structures of the communities. All other microbiome taxonomic composition analyzes were done using MicrobiomeAnalyst (Chong et al., 2020). Permutational multivariate analysis of variance (PERMANOVA) was implemented to identify significant differences between the tested groups (location/season). ‘DESeq2’ was used to find differentially abundant taxa between samples with a cut-off of adjusted value of *p* <0.05.

## Results

### Environmental parameters and microplastic profiles of the Yarkon (Mediterranean Sea) and Sado (Atlantic Ocean) collection sites

The basic water parameters at the time of sampling and the physical characteristics of the sampled microplastics from the Yarkon and Sado collection sites were compared between contrasting seasons. The water temperature and salinity during winter collections were 4.3 degrees and 5.2 practical salinity unit (PSU) higher in Yarkon, compared to Sado, and 11.8 degrees and 2.5 PSU higher during summer collections (Table 1). Between seasons, the temperatures varied in the Yarkon site (±10.2 degrees) and were less variable in the Sado site (±2.7 degrees). Salinity had the opposite trend and was less variable in the Yarkon site (±0.1 PSU) when compared to the Sado site (±0.6 PSU).

**Figure 2:**
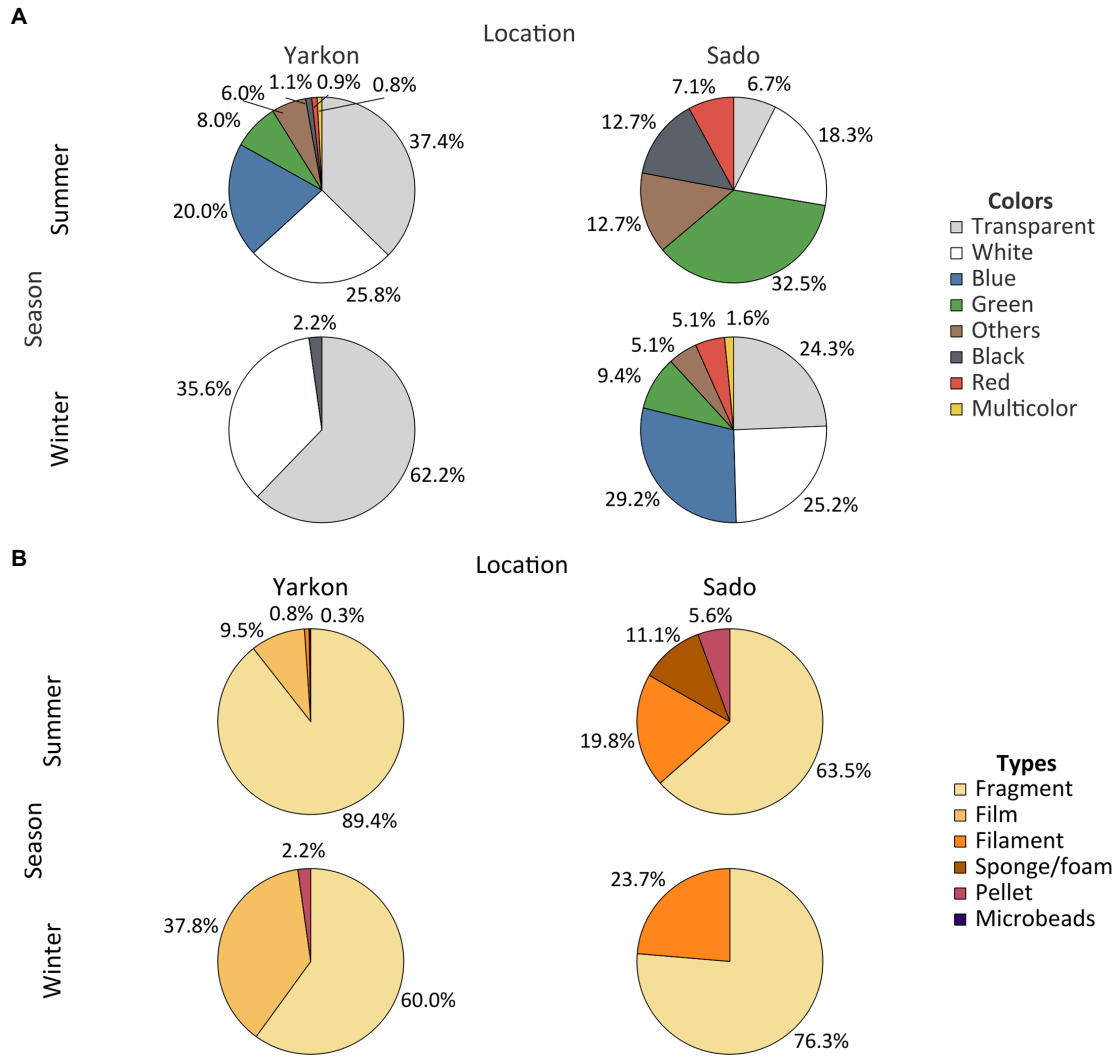
Physical characteristics of the microplastic. **(A)** Plastic abundances by color in both locations and seasons. **(B)** Abundance of plastic types in both locations and seasons. Results are expressed in % of polymers relative to total microplastics analyzed.

**Figure 3:**
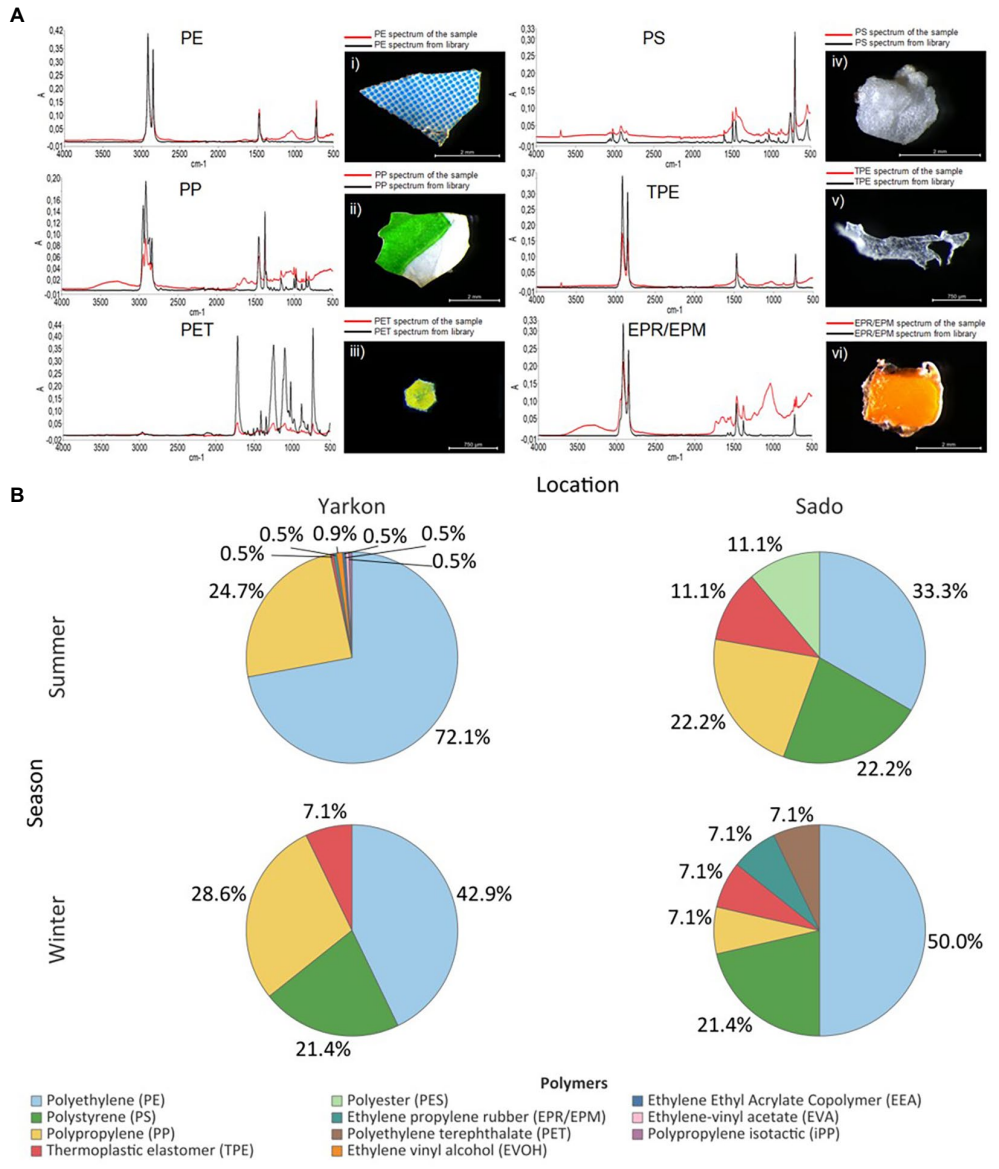
Microplastic polymer composition. **(A)** Infrared spectra of the most common polymers from Yarkon and Sado (red) and their reference spectra (black): (i) blue and white fragment with PE spectrum (Yarkon summer); (ii) white and green fragment (Yarkon summer) with PP spectrum; (iii) iridescent glitter particle with PET spectrum (Sado winter); (iv) a foam particle with PS spectrum (Sado summer); (v) TPE fragment and its spectrum (Sado summer); (vi) an orange fragment with EPR/EPM spectrum (Yarkon summer). The y-axis corresponds to absorbance–A, and the *x*-axis to wavenumber (cm-^1^). **(B)** Polymers composition in Yarkon and Sado according to season. Results are expressed in % of polymers relatively to the total microplastics analyzed.

The winter samples from the Yarkon, and all the samples from Sado contained similar concentrations of microplastic particles (between 0.04 and 0.08 particles m^−3^ on average), whereas the summer Yarkon samples contained significantly higher (*p*-value <0.05) microplastic particles concentrations (112.59 particles m^−3^ on average; Table 1; Supplementary Table S2). The color composition of the microplastic also varied between locations; Yarkon samples contained microplastic particles that were mostly white or transparent in both seasons, whereas the Sado samples showed higher diversity of colors with blue and green being the most abundant colors (Figure 2A; Supplementary Table S3). Differences between seasons were also found. Blue, green and brown microplastic particles were found in the winter samples and were absent from the summer samples from Yarkon. On the other hand, the Sado summer samples contained black microplastics and did not contain blue microplastics, opposite to the Sado winter samples. The most common shapes of plastic particles in both sites were fragments, however other typologies were found in variable abundances (Figure 2B; Supplementary Table S4). Samples from Yarkon registered a significantly higher percentage of films (*p*-value = 0.006; 37.8%), particularly during winter. On the other hand, Sado samples contained higher percentages of filaments (19.8–23.6%) as well as higher percentages of sponge/foam and pellets during the winter (11.1 and 5.6% respectively). Of the 854 potential plastic particles that were identified by a stereoscope, 252 polymers were analyzed using FTIR (~29.5% of all potential plastics). All the particles analyzed were identified as synthetic polymers. The spectra of the six most common polymers identified are shown in Figure 3A. PE was the most dominant polymer followed by PP or PS, depending on the location and season (Figure 3B). The Yarkon winter samples contained 21.4% of PS, which was absent from the summer samples, as well as higher percentages of thermoplastic elastomer (TPE; 7.1% vs. <0.5%) compared to the summer samples from the same location. The Sado winter samples contained higher percentages of PE (50% vs. 33.3% in the summer), PET (7.1% vs. 0% in the summer), and ethylene propylene rubber (EPR/EPM; 7.1% vs. 0% in the summer), whereas the summer samples consist of higher concentrations of PP (22.2% vs. 7.1% in the winter) and polyester (PES; 11.1% vs. 0% in the winter).

### Location is the strongest source of microplastic microbiome variation followed by season

**Figure 4:**
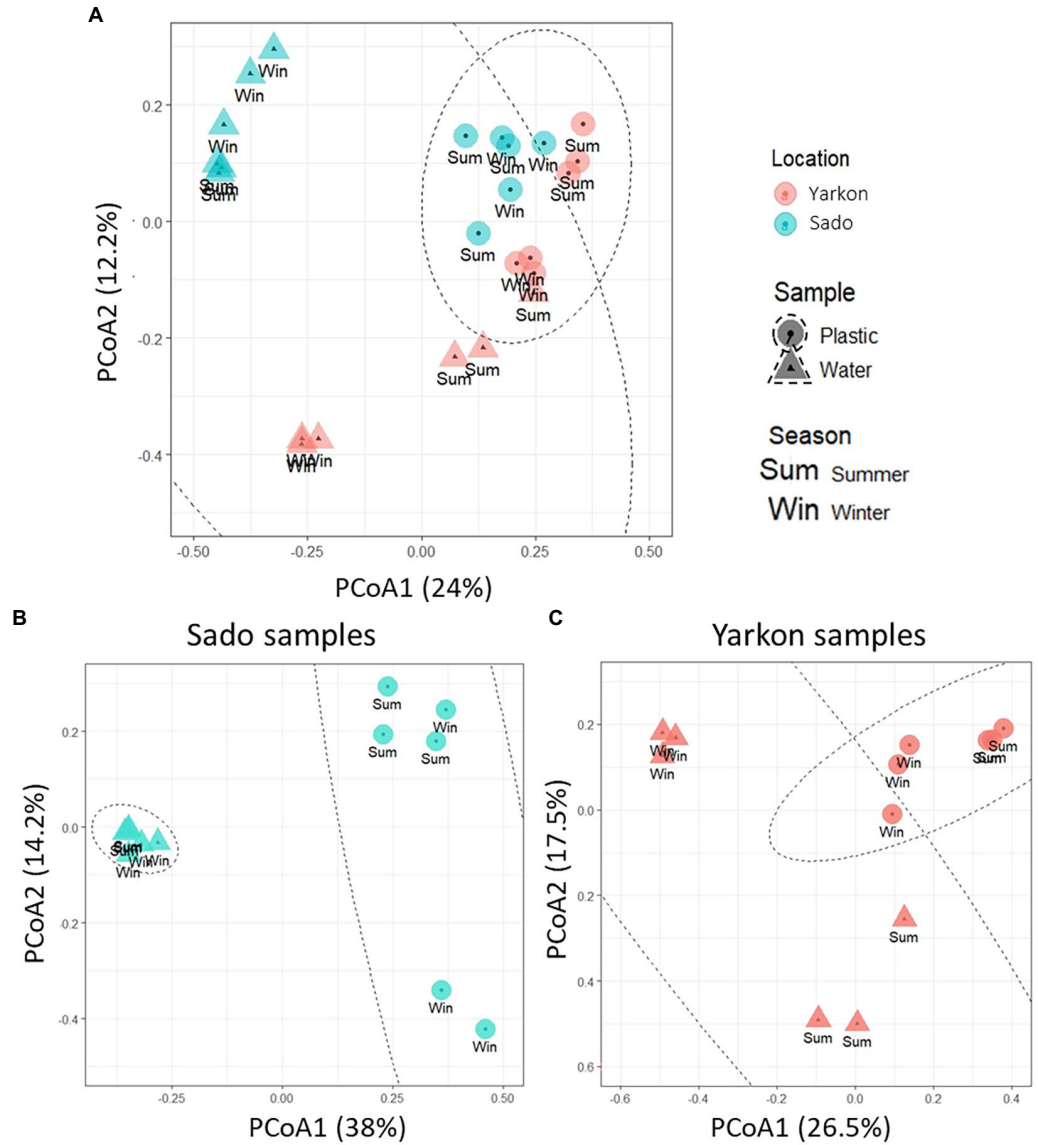
PCoA plot of the Bray-Curtis dissimilarities among the 16S-based microbiomes of the samples (Beta diversity, *N* = 3 per treatment). **(A)** Diversity among all samples. **(B)** Diversity among Sado samples. **(C)** Diversity among Yarkon samples.

The nanopore sequencing run produced an average of 12,408 reads per sample with an average read length of 1,388 bp and mapping rate of 99.5% (Supplementary Table S5). The community complexity analysis showed similar results for all samples in all of the alpha diversity indexes (richness and diversity; Supplementary Figure S2). On the other hand, the analysis of the diversity between samples (beta diversity) demonstrated a clear distinction (*p*-value ≤0.01), (mostly in component 1 which explained 24% of the variation) between the microplastic bacterial communities (circles) and those of the surrounding water (triangles; Figure 4A). The microplastic microbiome communities were further separated (*p*-value ≤ 0.01) according to location (Yarkon/Sado). A significant (*p*-value = 0.01) seasonal variation was observed only between summer and winter samples from Yarkon (Table 2).

**Table 2:** *P*-values of PERMANOVA comparisons performed on bacterial communities.

	Factor	*P-*value
Sample		**0.001**
Water	Location*	**0.003**
	Season**	0.116
Plastic	Location	**0.003**
	Season	0.050
Location		**0.001**
Yarkon	Sample***	**0.002**
	Season	**0.011**
Sado	Sample	**0.002**
	Season	0.107
Season		0.094

Significant results are emboldened (*p* < 0.05). * Yarkon vs Sado sites, ** winter vs summer. *** Water vs plastic.

To analyze the seasonal effect on the bacterial community composition in the Yarkon and Sado collection sites, PCoA analysis was separately performed for each location (Figures 4B,C). Both Yarkon microplastic and water samples clustered according to the seasons. Among the Sado samples (Figure 4B), the water samples showed high similarity (clustered) regardless of the season, whereas the microplastic samples were more variable (dispersed). In contrast, the water samples from Yarkon showed greater variation between seasons when compared with the microplastic samples.

### The plastisphere bacterial phyla

**Figure 5:**
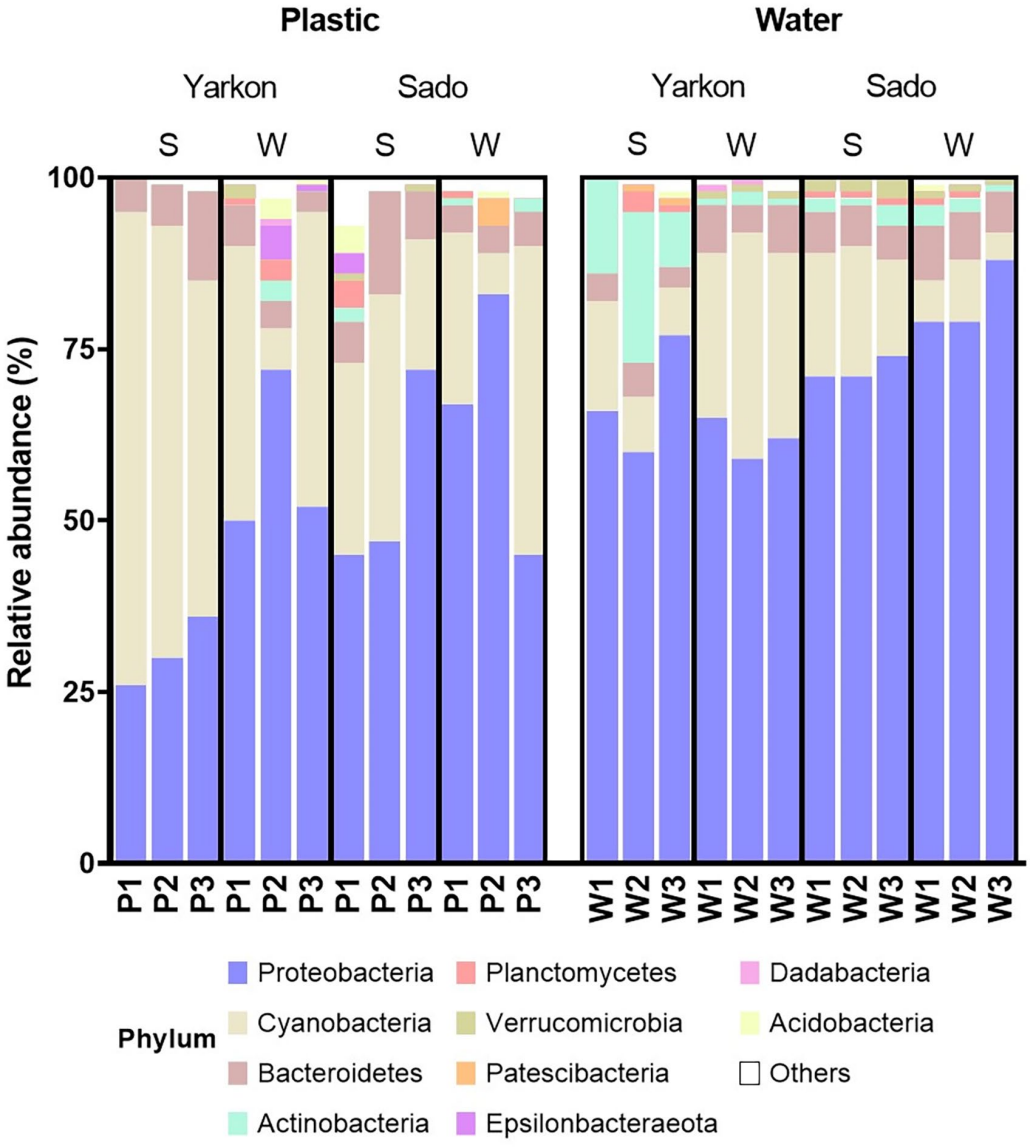
Relative abundances of the top 10 phyla in the microplastic and water samples from the Yarkon and Sado divided by seasons (S = summer, W = winter). P1-P3 represent repeats of the plastic samples, W1-W3 represent repeats of the water samples.

Proteobacteria and Cyanobacteria were the dominant phyla in the microbiomes of all samples (Figure 5). In general, the microplastic samples contained higher percentages of Cyanobacteria and lower percentages of Proteobacteria compared to the water samples. This could be seen most clearly in the Yarkon summer samples, with relative Cyanobacteria abundance of 49–69% in the microplastic samples, compared to only 7–16% in the equivalent water samples. Other differences in the phyla composition included higher percentages of Actinobacteria in the water samples, especially in the summer samples from Yarkon. Overall, the microplastic microbiome showed higher variability among repeats compared to the water samples. For example, the presence of Epsilonbacteraeota and Acidobacteria was observed in the Yarkon winter repeat 2 and Sado summer repeat 1, whereas all other samples contain low percentages of these phyla (Figure 5).

### Is there a marine plastic microbiome core?

**Figure 6:**
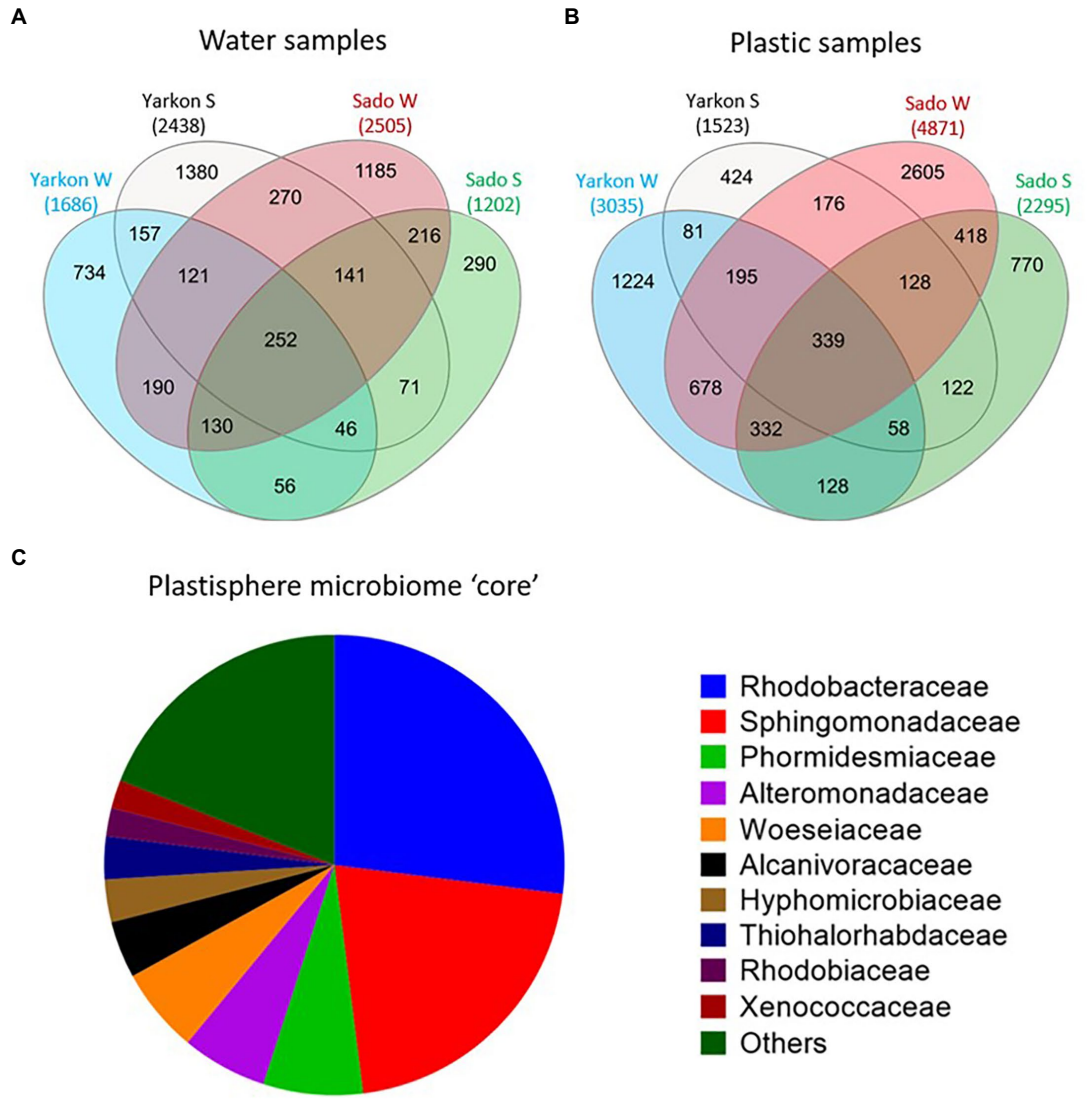
Plastic microbiome ‘core’. **(A)** Shared and unique OTUs among the locations and seasons for water and **(B)** plastic samples. **(C)** Microbial composition of the ‘core’ plastisphere microbiome at the family level (based on 246 of the 339 ‘core’ OTUs that were not found in the water ‘core’).

To further understand the taxonomic composition of the plastisphere microbiome across locations and seasons, we searched for a shared ‘core’ microbiome as well as for differentially abundant taxa. When comparing among samples with repeats combined, we identified 339 shared OTU in all microplastic samples compared with 252 in the water samples (Figures 6A,B). Of those OTUs, 93 were found in both cores and 246 OTUs that were specific to the plastic microbiome core. The plastic-specific 246 OTUs corresponded to 182 database matches classified within 41 families.

The 10 top families (Figure 6C) corresponded to 80% of the core and included *Rhodobacteraceae* (27%), *Sphingomonadaceae* (21%), *Phormidesmiaceae* (7%), *Alteromonadaceae* (6%), *Woeseiaceae* (6%), *Alcanivoracaceae* (4%), *Hyphomicrobiaceae* (3%), *Thiohalorhabdaceae* (3%), *Rhodobiaceae* (2%), and *Xenococcaceae* (2%). Only 29 bacteria were identified at the species level, including 19 from the family *Rhodobacteraceae*, 6 from *Sphingomonadaceae*, 2 from *Alteromonadaceae*, one from *Pseudomonadaceae*, and one from *Hyphomonadaceae* (Supplementary Table S6). All of the 29 identified species were typically rare with mean relative abundance of 0.01–0.06%. The species with the highest mean relative abundance was *Roseovarius aestuarii* with 0.21% of the total reads that reached as high as 0.42% in Israeli summer. When comparing all sample repeats without combining them together, we did not find any shared plastic-specific OTUs. This agrees with the low abundance of the bacteria that were found in the above comparison.

**Figure 7:**
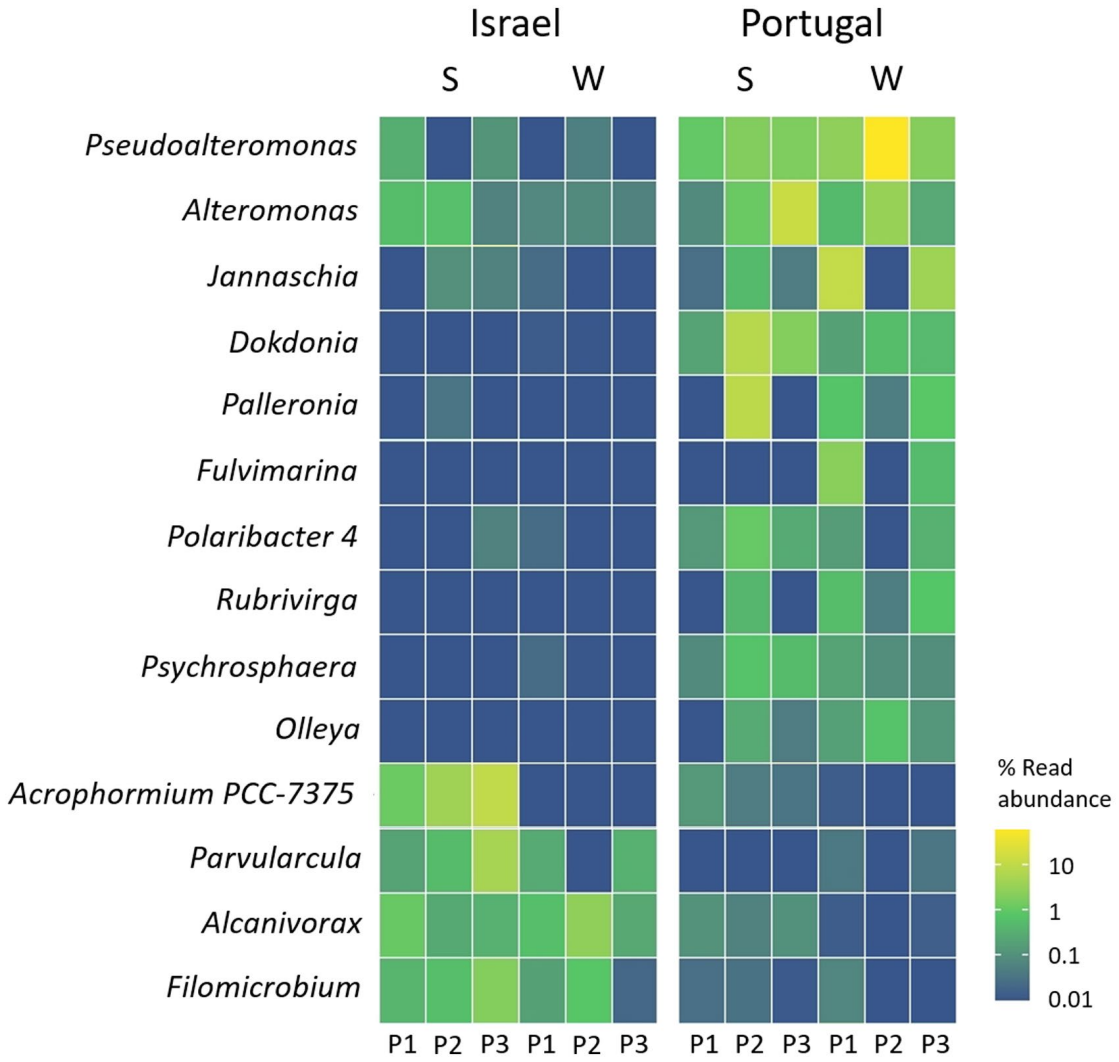
Genera in the plastic samples with significant higher relative mapped read abundance in one location over another (threshold–adjusted value of *p* (FDR) of <0.05). S = summer, W = winter.

We further looked into the taxa that were differentially abundant on the collected microplastic in either Yarkon or Sado sampling sites and identified 14 genera and 11 species that were significantly enriched in one location over the other. The genera *Acrophormium PCC-7375*, *Parvularcula*, *Filomicrobium*, and *Alcanivorax* were more abundant in Yarkon (Israel), while the genera *Pseudoalteromonas*, *Alteromonas*, *Jannaschia*, *Dokdonia*, *Palleronia*, *Fulvimarina*, *Polaribacter*, *Rubrivirga*, *Psychrosphaera*, and *Olleya* were more abundant in Sado (Portugal) (Figure 7; Supplementary Table S7). Among the species identified, only one species, *Loktanella* sp. *S4079,* was enriched in Yarkon, while the rest, including: *Alteromonas genovensis*, *Xenococcaceae cyanobacterium CENA315*, *Jannaschia faecimaris*, *Fulvimarina pelagi*, *uncultured gamma proteobacterium CHAB IV 34*, *Dokdonia* sp. *PRO95*, *Loktanella* sp. *S4079* and three species of *Pseudoalteromonas* (*BSw20060*, *13–15* and *Arctic P16*) were enriched in Sado (Supplementary Table S7).

### Potential pathogens

**Figure 8:**
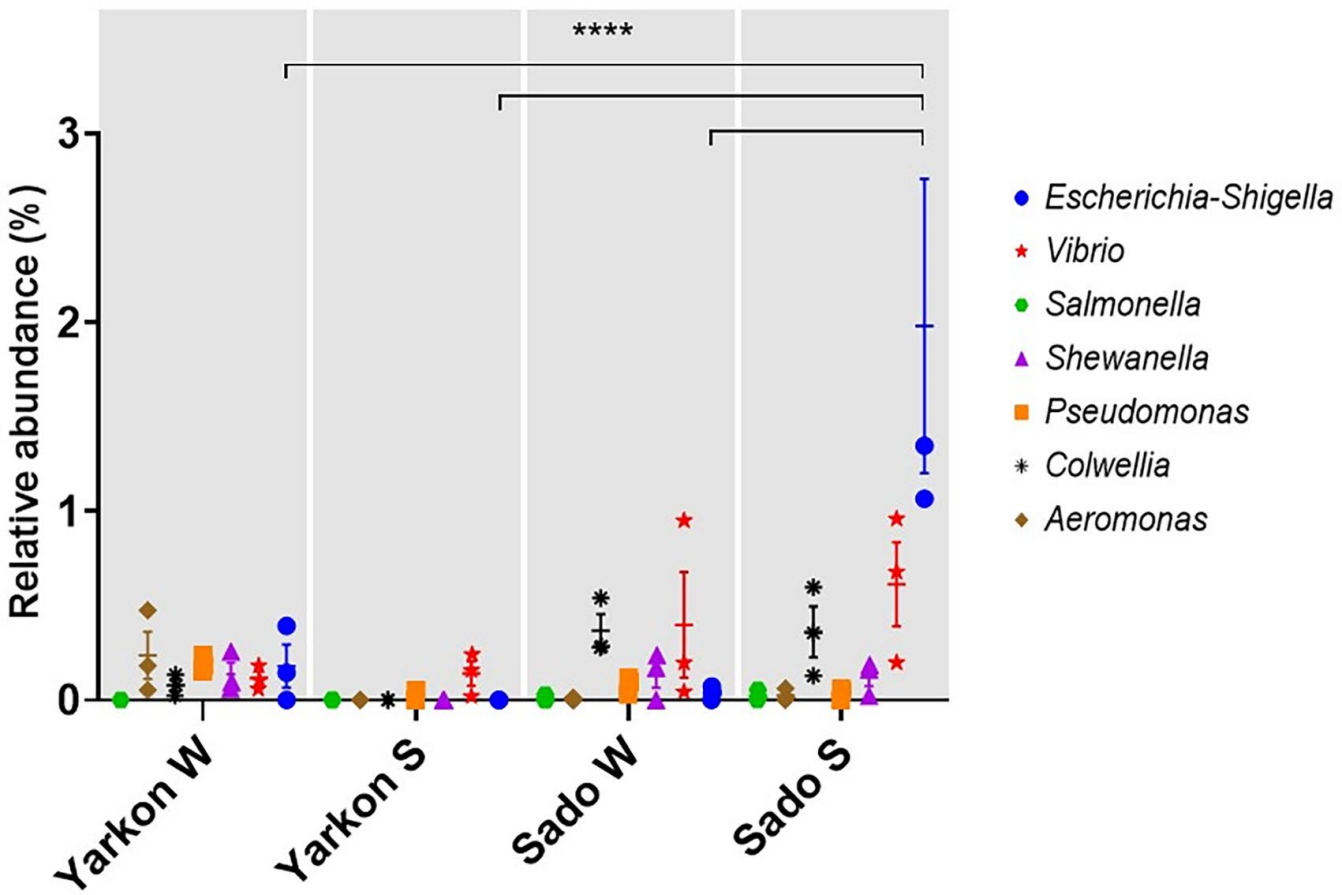
Relative abundances of potentially pathogenic genera in the microplastic samples from Yarkon and Sado in winter (W) and summer (S). Significance was assessed using 2-way ANOVA. *N* = 3. *p* = <0.0001.

The proximity of the chosen sampling sites to major river estuaries (Yarkon and Sado) enabled us to examine the effect of terrestrial anthropogenic contaminations. We found that the microplastic samples carried potentially pathogenic bacteria, including of the *Escherichia-Shigella*, *Vibrio*, *Salmonella*, *Shewanella*, *Pseudomonas*, *Colwellia*, and *Aeromonas* genera (Figure 8). Significantly higher abundance of *Escherichia-Shigella* genus was identified in the Sado summer microplastic samples compared to the other locations and seasons (Figure 8).

## Discussion

In this study, we analyzed and compared the physical and biological composition of microplastics collected from the Mediterranean Sea (Israel) and the Atlantic Ocean (Portugal) in two opposing seasons–summer and winter. The two locations chosen for this study were both in proximity to a river estuary (Yarkon in Israel and Sado in Portugal) where terrestrial inputs of plastic are expected to be the highest. Overall, our results suggest that the plastic microbiome is primarily affected by location and to a lesser extent by the season. The drivers for the geographical segregation are the parameters that vary between the sites, including the microplastic composition and the environmental biotic and abiotic conditions as well as anthropogenic influences. Two of the most important abiotic factors that may affect the plastisphere microbiome are temperature and salinity. Temperature and salinity are known to affect biofilm growth and diversity (Li et al., 2019, 2020). For example, warmer waters and higher salinity have been reported as important drivers of plastic-specific communities (Oberbeckmann et al., 2018). The differences in temperature and salinity may also be key drivers for the changes in the microbiome composition observed between the seasons (Pinnell and Turner, 2020; Zhang et al., 2021). Indeed, a bigger difference in the microplastic microbiome composition between winter and summer was observed in Yarkon, where seasonal difference in temperature was more than 10°C. A significant difference in microbiome composition between winter and summer was not seen in Sado, which suggests the influence of seasonal changes can vary depending on location. Other environmental parameters associated with seasonal changes include light intensity and the availability of nutrients (Pinto et al., 2019; Amaral-Zettler et al., 2020; Roth Rosenberg et al., 2021). Nutrient concentrations were not measured but also may have contributed to plastic specificity as it has been shown that low nutrient levels trigger surface attachment for many bacterial species (Stanley and Lazazzera, 2004; Oberbeckmann et al., 2018). Previous studies along the Israeli coast reported the lowest macronutrient concentrations in July (Roth Rosenberg et al., 2021).

Although it seems that environmental factors have the strongest influence on the plastisphere microbiome, another contributing factor may be the concentration and composition of the microplastic itself. Overall, the microplastic concentrations were similar among samples with the exception of the Yarkon summer samples (112.59 particles m^−3^). This significantly higher concentration is an outlier, as the average microplastic concentrations along the Israeli coast were reported to be 7.68 ± 2.38 particles m^−3^ (van der Hal et al., 2017). A similar outlier was recorded during the drier summer season in Neve Yam, Israel (324.1 particles m^−3^; van der Hal et al., 2017), which is located on the same coastline as our sampling spot near Yarkon estuary. These outliers in plastic concentrations may reflect patchiness in the distribution of plastic particles along the Israeli coast due to its unique geomorphology or local factors such as wind, swell, and currents causing its accumulation in specific areas. In Portugal, a recent study reported microplastic concentrations of 0.45 ± 0.52 particles m^−3^ in Arrábida and Setúbal coastal area (Rodrigues et al., 2022) that are proximal to Sado estuary. These concentrations are higher than those documented in our study near Sado estuary of 0.04 ± 0.02 particles m^−3^ and 0.08 ± 0.04 particles m^−3^ during summer and winter, respectively. The abundance of microplastics along the Portuguese coast, may be affected by the oceanographic characteristics of the western Iberian coast, such as seasonal upwelling, river plumes and currents of the Iberian Pole (IPC) and the Portugal current (Santos et al., 2011; Alves and Miranda, 2013).

Plastic particle colors varied between both locations and seasons. In Yarkon collection site the colors of plastics were more diverse during summer when compared to winter. In both seasons the predominant colors were transparent (37.4% in summer and 62.2% in winter) and white (25.8% in summer and 35.6% in winter). The predominance of light colors was also recorded by Van der Hal et al. (2017) along the Israeli Mediterranean coast in both summer and winter and by Marrone et al. (2021) in the central part of the Mediterranean Sea. In contrary to the microplastic color distribution in Yarkon samples, in the Sado samples the green particles (32.54%) were the most abundant in the summer; and the blue particles (29.23%) were the most abundant in the winter. Delacuvellerie et al. (2022) suggest that the color and size of plastics collected from the environment do not influence the bacterial biofilm structure. However, in an incubation experiment, Wen et al. (2020) showed clear separation between plastisphere communities on three colors of microplastics. The effect of the color on the microbiome composition may be due to light availability determined by a specific pigment or due to the chemistry of the dye material itself. Most polymer types have a number of different additives, including dyes, that are not covalently bound to the plastic and so leach out into the environment during aging reviewed in (Hahladakis et al., 2018) which can be an additional factor effecting the microplastic biome.

The most common plastic debris shapes in both locations were fragments. The predominance of fragments in both seasons in Yarkon, agrees with the most frequently found typologies along the Mediterranean coast of Israel (93.6–97.7%; van der Hal et al., 2017), Mediterranean basin (87.7%; Cózar et al., 2015) and in open and coastal waters of the eastern Mediterranean Sea (including the Ionian and Levantine seas; 50–60%; Adamopoulou et al., 2021). The high percentage of films documented in the winter (37.78%), can be explained by the faster degradation of plastic bags and packaging, which is considered their main source (Marrone et al., 2021). The same was observed for Sado, where fragments predominated during summer (63.49%) and winter (76.34%), which is in agreement with samples from the North East Atlantic (63%) and from Arrábida and Setúbal (Rodrigues et al., 2022).

The most abundant polymers identified in both locations were PE, PP, and PS, that are also the polymer types with the highest demand in Europe in 2019 and 2020 (Plastic Europe, 2021) and the most abundant in aquatic environments (Erni-Cassola et al., 2019). These polymers which are predominant in the upper layers of the water column (Andrady, 2011; Erni-Cassola et al., 2019) have lower densities than seawater, ranging from 0.89 to 0.98 g cm^−3^ (Zhou et al., 2021). In this study the microplastic debris were collected from the water surface where these floating polymers are abundant (Pasquier et al., 2022). Although PET buoyancy is usually negative and therefore it is expected to be found in the sediment (Lenaker et al., 2019), its appearance in the Portugal winter samples may be due to the heavy rainfall, typical to this season, that may lead to strong currents and the uplifting of sediment. Moreover, land-based polyester fibers, which were also identified in the samples, may arrive to the sea from wastewater or water run-offs and may not be heavy enough to break the surface tension of the water. The prevalence of PE, PP and PS polymers in Yarkon, is in line with reports from the Ionian, Aegean, and Levantine Seas (Adamopoulou et al., 2021), central Mediterranean Sea (Marrone et al., 2021) and other Mediterranean Sea studies (Suaria et al., 2016; Sharma et al., 2021). Similar to Yarkon, PE and PP polymers were also the most common in Sado, which agrees with studies in Arrábida and Setúbal (Rodrigues et al., 2022). The plastic polymer type is not considered a major factor that is affecting the plastisphere microbiome in the natural environment (Oberbeckmann et al., 2018; Laverty et al., 2020). However, short-term incubation studies have shown that some bacteria have a significant preference toward specific plastic polymers (Marsay et al., 2022).

Additional anthropogenic factors may influence the composition of the plastisphere microbiome, especially in proximity to estuaries. In this study, we analyzed the relative abundances of seven microbial genera that include strands associated with human diseases. The most prominent among them were *Escherichia-Shigella*, *Vibrio,* and *Colwellia* that were relatively more abundant in the Sado samples compared to those collected in Yarkon. *Escherichia-Shigella* genus is often associated with human and animal pathogens and considered an indicator of fecal contamination (Garcia-Aljaro et al., 2019). The relative abundance of *Escherichia-Shigella* was significantly higher in Sado during summer compared to the winter and to Yarkon which may be related to either differences in river/runoff flows or in waste water inputs. We would like to note that with the absence of other assays to test virulence, presence of relevant genes or toxins, this kind of analysis is not sufficient to draw any conclusions on the pathogenicity of the mentioned genera. However, the analysis provides us with yet another evidence for the role of marine microplastic as a stable vector for anthropogenic biological contaminations.

To understand the extent of geographical and seasonal variability in the plastisphere composition in this study, we further investigated the taxonomic content of the plastisphere microbiome using MinION nanopore sequencing platform and a tailored bioinformatics pipeline. We previously demonstrated that this method provides high-resolution taxonomic data, often up to the species level (Davidov et al., 2020). At the phylum level, Cyanobacteria were highly enriched on the microplastic surfaces compared to the surrounding water while Proteobacteria showed the opposite trend. As photosynthetic bacteria, Cyanobacteria may benefit from constant higher sunlight exposure when attached to floating plastic debris. This trend was especially noticed in the Israeli summer, which is characterized with clear sky and increased sunlight radiation. Similar to our results, Cyanobacteria were previously recorded to be dominant on marine microplastics (Bryant et al., 2016). In contrary, Actinobacteria and Verrucomicrobia were more abundant in the water samples compared to the microplastic samples (Wright et al.,2020).

One of the questions addressed in this study is the the existence of a common plastisphere microbial ‘core’. It has been proposed that the ‘core’ microbiome differs depending on season, geography, plastic type (Kirstein et al., 2018; Roager and Sonnenschein, 2019; Amaral-Zettler et al., 2020), or biofilm development stage (Tu et al., 2020). Moreover, the identification and characterization of such a ‘core’ is dependent on its definition. When searching for shared plastic-specific OTUs with three repeats combined (pooled) for each location/season, we identified as many as 246 OTUs that corresponded to 182 species/strains that were classified within 41 families. However, when regarding each of the repeats as a separated sample, we did not find even one shared OTU. This result is similar to a previous report that showed that no ASVs were shared among all plastic samples between locations near cities on the west and east of Italy (Basili et al., 2020). Another study that found a “core” plastic microbiome containing only 26 shared OTUs reported very low relative abundances for these strains (<0.1%; Scales et al., 2021). At higher taxonomic levels, different studies find different families or genera depending on the environment or the method used to establish a ‘core’ (De Tender et al., 2017; Jiang et al., 2018; Kesy et al., 2019).

When searching for differentially abundant low-level taxa between locations, we identified 10 genera that were significantly enriched in Sado Vs. only 4 that were enriched in Yarkon. Among the latter, it is noteworthy to mention the genus *Alcanivorax* that showed high abundance in all of the Yarkon repeats and was also identified in lower abundance in Sado summer samples, suggesting that this genus is favorable of higher temperatures. In two separated incubation experiments, we previously identified substantial *Alcanivorax* presence in association with PE surfaces (Davidov et al., 2020; Marsay et al., 2022). *Alcanivorax* species are well-known degraders of alkanes and petroleum (Hara et al., 2003). It was also recently shown that *Alcanivorax* sp. 24 species is able to break-down weathered low-density PE and use it as a nutritional carbon source (Zadjelovic et al., 2022). There was an oil spill that polluted Israel’s coast at the end of February 2021. However, our sampling took place in the middle of July 2021 almost 5 months later, and therefore, we assume that the results are not directly affected by this local event.

Supported by previous studies, the results of this study suggest that although different plastispheres share common features, the microbial content varies greatly according to the geographic location, biotic and abiotic conditions in the marine environment and local anthropogenic factors, and therefore may contain a very limited microbiome “core.” In that sense, it may be more accurate to perceive the plastisphere as multiple different ecosystems rather than a single one. Accordingly, additional knowledge may be gained by further exploring the plastisphere in different geographical locations and climates with seasonal sampling that is repeated over more than 1 year.

## Data availability statement

The datasets presented in this study can be found in online repositories. The names of the repository/repositories and accession number(s) can be found in the article/Supplementary material.

## Author contributions

KM, AA, MM, NF, SI, and KD did the bench work. KM, AA, IY, and MO performed the field work. KM, MO, and AA wrote the main manuscript. YK and KM did the bioinformatic analyzes. MO and PS discussed, reviewed, edited the manuscript and received the funding. All authors contributed to the article and approved the submitted version.

## Funding

This work was supported by the Israel Ministry of Science and Technology Israel-Portugal collaboration grant (3–1650) and the FCT-Foundation for Science and Technology (Portugal), FCT/MCTES (PT-IL/0001/2019), and through the strategic project UIDB/04292/2020 granted to MARE and under the project LA/P/0069/2020 granted to the Associate Laboratory ARNET.

## Conflict of interest

The authors declare that the research was conducted in the absence of any commercial or financial relationships that could be construed as a potential conflict of interest.

## Publisher’s note

All claims expressed in this article are solely those of the authors and do not necessarily represent those of their affiliated organizations, or those of the publisher, the editors and the reviewers. Any product that may be evaluated in this article, or claim that may be made by its manufacturer, is not guaranteed or endorsed by the publisher.

## Footnotes

^1^https://www.ncbi.nlm.nih.gov/sra

## Supplementary material

The Supplementary material for this article can be found at: https://www.frontiersin.org/articles/10.3389/fmicb.2023.1089926/full#supplementary-material

## References

[ref1] Adamopoulou A. Zeri C. Garaventa F. Gambardella C. Ioakeimidis C. Pitta E. (2021). Distribution patterns of floating microplastics in open and coastal waters of the eastern Mediterranean Sea (Ionian, Aegean, and Levantine seas). Front. Mar. Sci. 8:699000. doi: 10.3389/fmars.2021.699000

[ref2] Alves J. M. R. Miranda P. M. A. (2013). Variability of Iberian upwelling implied by ERA-40 and ERA-interim reanalyses. Tellus A Dyn. Meteorol. Oceanogr. 65:19245. doi: 10.3402/tellusa.v65i0.19245

[ref3] Amaral-Zettler L. A. Zettler E. R. Mincer T. J. (2020). Ecology of the plastisphere. Nat. Rev. Microbiol. 18, 139–151. doi: 10.1038/s41579-019-0308-0 31937947

[ref4] Amaral-Zettler L. A. Zettler E. R. Slikas B. Boyd G. D. Melvin D. W. Morrall C. E.. (2015). The biogeography of the Plastisphere: implications for policy. Front. Ecol. Environ. 13, 541–546. doi: 10.1890/150017

[ref5] Andrady A. L. (2011). Microplastics in the marine environment. Mar. Pollut. Bull. 62, 1596–1605. doi: 10.1016/j.marpolbul.2011.05.030 21742351

[ref6] Basili M. Quero G. M. Giovannelli D. Manini E. Vignaroli C. Avio C. G.. (2020). Major role of surrounding environment in shaping biofilm community composition on marine plastic debris. Front. Mar. Sci. 7:262. doi: 10.3389/fmars.2020.00262

[ref7] Bolyen E. Rideout J. R. Dillon M. R. Bokulich N. A. Abnet C. C. Al-Ghalith G. A.. (2019). Reproducible, interactive, scalable and extensible microbiome data science using QIIME 2. Nat. Biotechnol. 37, 852–857. doi: 10.1038/s41587-019-0209-9, PMID: 31341288 PMC7015180

[ref8] Borrelle S. B. Ringma J. Law K. L. Monnahan C. C. Lebreton L. McGivern A.. (2020). Predicted growth in plastic waste exceeds efforts to mitigate plastic pollution. Science 369, 1515–1518. doi: 10.1126/science.aba3656, PMID: 32943526

[ref9] Bryant J. A. Clemente T. M. Viviani D. A. Fong A. A. Thomas K. A. Kemp P.. (2016). Diversity and activity of communities inhabiting plastic debris in the North Pacific gyre. MSystems 1:3. doi: 10.1128/msystems.00024-16, PMID: 27822538 PMC5069773

[ref10] Chong J. Liu P. Zhou G. Xia J. (2020). Using microbiome analyst for comprehensive statistical, functional, and meta-analysis of microbiome data. Nat. Protoc. 15, 799–821. doi: 10.1038/s41596-019-0264-1, PMID: 31942082

[ref11] Coates J. (2006). “Interpretation of infrared spectra, a practical approach” in Encyclopedia of Analytical Chemistry: Applications, Theory and Instrumentation. ed. Meyers R. A. (Chichester, UK: John Wiley & Sons, Ltd.), 10815–10837.

[ref001] Cole M. Lindeque P. Halsband C. Galloway T. S. (2011). Microplastics as contaminants in the marine environment: A review. Marine Pollution Bulletin. 62, 2588–2597. doi: 10.1016/j.marpolbul.2011.09.025 22001295

[ref12] Coons A. K. Busch K. Lenz M. Hentschel U. Borchert E. (2021). Biogeography rather than substrate type determines bacterial colonization dynamics of marine plastics. PeerJ 9:e12135. doi: 10.7717/peerj.12135, PMID: 34603853 PMC8445087

[ref13] Cózar A. Martí E. Duarte C. M. García-de-Lomas J. van Sebille E. Ballatore T. J.. (2017). The Arctic Ocean as a dead end for floating plastics in the North Atlantic branch of the Thermohaline circulation. Sci. Adv. 3:e1600582. doi: 10.1126/sciadv.1600582, PMID: 28439534 PMC5397136

[ref14] Cózar A. Sanz-Martín M. Martí E. González-Gordillo J. I. Ubeda B. Gálvez J. Á.. (2015). Plastic accumulation in the Mediterranean Sea. PLoS One 10:e0121762. doi: 10.1371/journal.pone.0121762, PMID: 25831129 PMC4382178

[ref15] Cunningham E. M. Ehlers S. M. Dick J. T. A. Sigwart J. D. Linse K. Dick J. J.. (2020). High abundances of microplastic pollution in Deep-Sea sediments: evidence from Antarctica and the Southern Ocean. Environ. Sci. Technol. 54, 13661–13671. doi: 10.1021/acs.est.0c03441, PMID: 33086791

[ref16] Davidov K. Iankelevich-Kounio E. Yakovenko I. Koucherov Y. Rubin-Blum M. Oren M. (2020). Identification of plastic-associated species in the Mediterranean Sea using DNA metabarcoding with Nanopore MinION. Sci. Rep. 10, 17533–17511. doi: 10.1038/s41598-020-74180-z, PMID: 33067509 PMC7568539

[ref17] De Tender C. Devriese L. I. Haegeman A. Maes S. Vangeyte J. Cattrijsse A.. (2017). Temporal dynamics of bacterial and fungal colonization on plastic debris in the North Sea. Environ. Sci. Technol. 51, 7350–7360. doi: 10.1021/acs.est.7b00697, PMID: 28562015

[ref18] Delacuvellerie A. Ballerini T. Frère L. Matallana-Surget S. Dumontet B. Wattiez R. (2022). From rivers to marine environments: a constantly evolving microbial community within the plastisphere. Mar. Pollut. Bull. 179:113660. doi: 10.1016/j.marpolbul.2022.113660, PMID: 35460946

[ref19] Dong X. Zhu L. Jiang P. Wang X. Liu K. Li C.. (2021). Seasonal biofilm formation on floating microplastics in coastal waters of intensified marinculture area. Mar. Pollut. Bull. 171:112914. doi: 10.1016/j.marpolbul.2021.112914, PMID: 34488149

[ref20] Erni-Cassola G. Zadjelovic V. Gibson M. I. Christie-Oleza J. A. (2019). Distribution of plastic polymer types in the marine environment; a meta-analysis. J. Hazard. Mater. 369, 691–698. doi: 10.1016/j.jhazmat.2019.02.067 30826562

[ref21] Garcia-Aljaro C. Momba M. Muniesa M. (2019). “Pathogenic members of *Escherichia coli* & *Shigella* spp. shigellosis” in Water and Sanitation for the 21st Century: Health and Microbiological Aspects of Excreta and Wastewater Management (Global Water Pathogen Project). eds. Pruden A. Ashbolt N. Miller J. (Michigan: Michigan State University).

[ref22] Hahladakis J. N. Velis C. A. Weber R. Iacovidou E. Purnell P. (2018). An overview of chemical additives present in plastics: migration, release, fate and environmental impact during their use, disposal and recycling. J. Hazard. Mater. 344, 179–199. doi: 10.1016/j.jhazmat.2017.10.014, PMID: 29035713

[ref23] Hara A. Syutsubo K. Harayama S. (2003). Alcanivorax which prevails in oil-contaminated seawater exhibits broad substrate specificity for alkane degradation. Environ. Microbiol. 5, 746–753. doi: 10.1046/j.1468-2920.2003.00468.x, PMID: 12919410

[ref24] Hummel D. O.. (2002). Atlas of Plastics Additives: Analysis by Spectrometric Methods. 125. Springer-Verlag, University of Cologne, Berlin, Germany, 1441.

[ref25] Jiang P. Zhao S. Zhu L. Li D. (2018). Microplastic-associated bacterial assemblages in the intertidal zone of the Yangtze estuary. Sci. Total Environ. 624, 48–54. doi: 10.1016/j.scitotenv.2017.12.105, PMID: 29247904

[ref26] Jung M. R. Horgen F. D. Orski S. V. Rodriguez C. V. Beers K. L. Balazs G. H.. (2018). Validation of ATR FT-IR to identify polymers of plastic marine debris, including those ingested by marine organisms. Mar. Pollut. Bull. 127, 704–716. doi: 10.1016/j.marpolbul.2017.12.061, PMID: 29475714 PMC13077791

[ref27] Karamitros T. Magiorkinis G. (2018). Multiplexed targeted sequencing for Oxford Nanopore MinION: a detailed library preparation procedure. Methods Mol. Biol. 1712, 43–51. doi: 10.1007/978-1-4939-7514-3_4 29224067

[ref28] Kesy K. Oberbeckmann S. Kreikemeyer B. Labrenz M. (2019). Spatial environmental heterogeneity determines young biofilm assemblages on microplastics in Baltic Sea Mesocosms. Front. Microbiol. 10:1665. doi: 10.3389/fmicb.2019.01665, PMID: 31447791 PMC6696623

[ref29] Kirstein I. V. Wichels A. Krohne G. Gerdts G. (2018). Mature biofilm communities on synthetic polymers in seawater - specific or general? Mar. Environ. Res. 142, 147–154. doi: 10.1016/j.marenvres.2018.09.028 30337052

[ref30] Lacerda A. L. D. F. Rodrigues L. D. S. van Sebille E. Rodrigues F. L. Ribeiro L. Secchi E. R.. (2019). Plastics in sea surface waters around the Antarctic Peninsula. Sci. Rep. 9:3977. doi: 10.1038/s41598-019-40311-4, PMID: 30850657 PMC6408452

[ref31] Laverty A. L. Primpke S. Lorenz C. Gerdts G. Dobbs F. C. (2020). Bacterial biofilms colonizing plastics in estuarine waters, with an emphasis on vibrio spp. and their antibacterial resistance. PLoS One 15:e0237704. doi: 10.1371/journal.pone.0237704, PMID: 32804963 PMC7430737

[ref32] Lenaker P. L. Baldwin A. K. Corsi S. R. Mason S. A. Reneau P. C. Scott J. W. (2019). Vertical distribution of microplastics in the water column and surficial sediment from the Milwaukee River basin to Lake Michigan. J. Environ. Sci. Technol. 53, 12227–12237. doi: 10.1021/acs.est.9b03850, PMID: 31618011

[ref33] Li J. Huang W. Jiang R. Han X. Zhang D. Zhang C. (2020). Are bacterial communities associated with microplastics influenced by marine habitats? Sci. Total Environ. 733:139400. doi: 10.1016/j.scitotenv.2020.139400, PMID: 32446095

[ref34] Li W. Zhang Y. Wu N. Zhao Z. Xu W. Ma Y.. (2019). Colonization characteristics of bacterial communities on plastic debris influenced by environmental factors and polymer types in the Haihe estuary of Bohai Bay. China Environ. Sci. Technol. 53, 10763–10773. doi: 10.1021/acs.est.9b03659, PMID: 31441645

[ref35] Lincoln S. Andrews B. Birchenough S. N. R. Chowdhury P. Engelhard G. H. Harrod O.. (2022). Marine litter and climate change: inextricably connected threats to the world’s oceans. Sci. Total Environ. 837:155709. doi: 10.1016/j.scitotenv.2022.155709, PMID: 35525371

[ref36] Mai L. Sun X.-F. Xia L.-L. Bao L.-J. Liu L.-Y. Zeng E. Y. (2020). Global riverine plastic outflows. Environ. Sci. Technol. 54, 10049–10056. doi: 10.1021/acs.est.0c02273, PMID: 32700904

[ref37] Marrone A. La Russa M. F. Randazzo L. La Russa D. Cellini E. Pellegrino D. (2021). Microplastics in the Center of Mediterranean: comparison of the two Calabrian coasts and distribution from coastal areas to the Open Sea. Int. J. Environ. Res. Public Health 18:10712. doi: 10.3390/ijerph182010712, PMID: 34682461 PMC8535489

[ref38] Marsay K. S. Koucherov Y. Davidov K. Iankelevich-Kounio E. Itzahri S. Salmon-Divon M.. (2022). High-resolution screening for marine prokaryotes and eukaryotes with selective preference for polyethylene and polyethylene terephthalate surfaces. Front. Microbiol. 13:845144. doi: 10.3389/fmicb.2022.845144, PMID: 35495680 PMC9042255

[ref39] McMurdie P. J. Holmes S. (2013). Phyloseq: an R package for reproducible interactive analysis and graphics of microbiome census data. PLoS One 8:e61217. doi: 10.1371/journal.pone.0061217, PMID: 23630581 PMC3632530

[ref40] Misic C. Covazzi Harriague A. (2019). Development of marine biofilm on plastic: ecological features in different seasons, temperatures, and light regimes. Hydrobiologia 835, 129–145. doi: 10.1007/s10750-019-3934-7

[ref41] Oberbeckmann S. Kreikemeyer B. Labrenz M. (2018). Environmental factors support the formation of specific bacterial assemblages on microplastics. Front. Microbiol. 8:2709. doi: 10.3389/fmicb.2017.02709, PMID: 29403454 PMC5785724

[ref42] Oberbeckmann S. Loeder M. G. J. Gerdts G. Osborn M. A. (2014). Spatial and seasonal variation in diversity and structure of microbial biofilms on marine plastics in northern European waters. FEMS Microbiol. Ecol. 90, 478–492. doi: 10.1111/1574-6941.12409, PMID: 25109340

[ref43] Oberbeckmann S. Osborn A. M. Duhaime M. B. (2016). Microbes on a bottle: substrate, season and geography influence community composition of microbes colonizing marine plastic debris. PLoS One 11:e0159289. doi: 10.1371/journal.pone.0159289, PMID: 27487037 PMC4972250

[ref44] Pagter E. Frias J. Nash R. (2018). Microplastics in Galway Bay: a comparison of sampling and separation methods. Mar. Pollut. Bull. 135, 932–940. doi: 10.1016/j.marpolbul.2018.08.013, PMID: 30301118

[ref45] Pasquier G. Doyen P. Kazour M. Dehaut A. Diop M. Duflos G.. (2022). Manta net: the Golden method for sampling surface water microplastics in aquatic environments. Front. Environ. Sci. 10:811112. doi: 10.3389/fenvs.2022.811112

[ref46] Peeken I. Primpke S. Beyer B. Gütermann J. Katlein C. Krumpen T.. (2018). Arctic Sea ice is an important temporal sink and means of transport for microplastic. Nat. Commun. 9:1505. doi: 10.1038/s41467-018-03825-5, PMID: 29692405 PMC5915590

[ref47] Pinnell L. J. Turner J. W. (2020). Temporal changes in water temperature and salinity drive the formation of a reversible plastic-specific microbial community. FEMS Microbiol. Ecol. 96:230. doi: 10.1093/femsec/fiaa230, PMID: 33181829

[ref48] Pinto M. Langer T. M. Hüffer T. Hofmann T. Herndl G. J. (2019). The composition of bacterial communities associated with plastic biofilms differs between different polymers and stages of biofilm succession. PLoS One 14:e0217165. doi: 10.1371/journal.pone.0217165, PMID: 31166981 PMC6550384

[ref49] Plastic Europe. (2021). *Plastics–The Facts 2021*. Plastic Europe, pp. 1–34. Available at: https://www.plasticseurope.org/en/resources/publications/4312-plastics-facts-2020 (Accessed May 10, 2022).

[ref50] Quast C. Pruesse E. Yilmaz P. Gerken J. Schweer T. Yarza P.. (2013). The SILVA ribosomal RNA gene database project: improved data processing and web-based tools. Nucleic Acids Res. 41, D590–D596. doi: 10.1093/nar/gks1219, PMID: 23193283 PMC3531112

[ref51] R Core Team. (2018). *R: A Language and Environment for Statistical Computing*. Vienna, Austria: R Foundation for Statistical Computing. Available at: https://www.r-project.org/.

[ref52] Roager L. Sonnenschein E. C. (2019). Bacterial candidates for colonization and degradation of marine plastic debris. Environ. Sci. Technol. 53, 11636–11643. doi: 10.1021/acs.est.9b02212, PMID: 31557003

[ref53] Rodrigues D. Antunes J. Pais J. Pequeno J. Caetano P. S. Rocha F.. (2022). Distribution patterns of microplastics in subtidal sediments from the Sado river estuary and the Arrábida marine park, Portugal. Front. Environ. Sci. 10:998513. doi: 10.3389/fenvs.2022.998513

[ref54] Roth Rosenberg D. Haber M. Goldford J. Lalzar M. Aharonovich D. Al-Ashhab A.. (2021). Particle-associated and free-living bacterial communities in an oligotrophic sea are affected by different environmental factors. Environ. Microbiol. 23, 4295–4308. doi: 10.1111/1462-2920.15611, PMID: 34036706

[ref55] Santos F. Gómez-Gesteira M. de Castro M. Álvarez I. (2011). Upwelling along the western coast of the Iberian Peninsula: dependence of trends on fitting strategy. Clim. Res. 48, 213–218. doi: 10.3354/cr00972

[ref56] Scales B. S. Cable R. N. Duhaime M. B. Gerdts G. Fischer F. Fischer D.. (2021). Cross-hemisphere study reveals geographically ubiquitous, plastic-specific bacteria emerging from the rare and unexplored biosphere. Clin. Vaccine Immunol. 6:e0085120. doi: 10.1128/mSphere.00851-20, PMID: 34106771 PMC8265672

[ref57] Sharma S. Sharma V. Chatterjee S. (2021). Microplastics in the Mediterranean Sea: sources, pollution intensity, sea health, and regulatory policies. Front. Mar. Sci. 8:494. doi: 10.3389/fmars.2021.634934

[ref58] Stanley N. R. Lazazzera B. A. (2004). Environmental signals and regulatory pathways that influence biofilm formation. Mol. Microbiol. 52, 917–924. doi: 10.1111/j.1365-2958.2004.04036.x, PMID: 15130114

[ref59] Suaria G. Avio C. G. Mineo A. Lattin G. L. Magaldi M. G. Belmonte G.. (2016). The Mediterranean plastic soup: synthetic polymers in Mediterranean surface waters. Sci. Rep. 6:1. doi: 10.1038/srep37551, PMID: 27876837 PMC5120331

[ref60] Tu C. Chen T. Zhou Q. Liu Y. Wei J. Waniek J. J.. (2020). Biofilm formation and its influences on the properties of microplastics as affected by exposure time and depth in the seawater. Sci. Total Environ. 734:139237. doi: 10.1016/j.scitotenv.2020.139237, PMID: 32450399

[ref61] Uddin S. Fowler S. W. Behbehani M. (2020). An assessment of microplastic inputs into the aquatic environment from wastewater streams. Mar. Pollut. Bull. 160:111538. doi: 10.1016/j.marpolbul.2020.111538, PMID: 32891961

[ref62] van der Hal N. Ariel A. Angel D. L. (2017). Exceptionally high abundances of microplastics in the oligotrophic Israeli Mediterranean coastal waters. Mar. Pollut. Bull. 116, 151–155. doi: 10.1016/j.marpolbul.2016.12.052, PMID: 28063700

[ref63] Weisburg W. G. Barns S. M. Pelletier D. A. Lane D. J. (1991). 16S ribosomal DNA amplification for phylogenetic study. J. Bacteriol. 173, 697–703. doi: 10.1128/jb.173.2.697-703.1991, PMID: 1987160 PMC207061

[ref64] Wen B. Liu J.-H. Zhang Y. Zhang H.-R. Gao J.-Z. Chen Z.-Z. (2020). Community structure and functional diversity of the plastisphere in aquaculture waters: does plastic color matter? Sci. Total Environ. 740:140082. doi: 10.1016/j.scitotenv.2020.140082, PMID: 32927571

[ref65] Wright R. J. Erni-Cassola G. Zadjelovic V. Latva M. Christie-Oleza J. A. (2020). Marine plastic debris: a new surface for microbial colonization. Environ. Sci. Technol. 54, 11657–11672. doi: 10.1021/acs.est.0c02305, PMID: 32886491

[ref66] Yang G. Gong M. Mai L. Zhuang L. Zeng E. Y. (2021). Diversity and structure of microbial biofilms on microplastics in riverine waters of the Pearl River Delta. China Chemosphere 272:129870. doi: 10.1016/j.chemosphere.2021.129870, PMID: 33607493

[ref67] Zadjelovic V. Erni-Cassola G. Obrador-Viel T. Lester D. Eley Y. Gibson M. I.. (2022). A mechanistic understanding of polyethylene biodegradation by the marine bacterium *Alcanivorax*. J. Hazard. Mater. 436:129278. doi: 10.1016/j.jhazmat.2022.129278, PMID: 35739790

[ref68] Zettler E. R. Mincer T. J. Amaral-Zettler L. A. (2013). Life in the ‘Plastisphere’: microbial communities on plastic marine debris. Environ. Sci. Technol. 47, 7137–7146. doi: 10.1021/es401288x, PMID: 23745679

[ref69] Zhang B. Yang X. Liu L. Chen L. Teng J. Zhu X.. (2021). Spatial and seasonal variations in biofilm formation on microplastics in coastal waters. Sci. Total Environ. 770:145303. doi: 10.1016/j.scitotenv.2021.145303, PMID: 33515883

[ref70] Zhang S.-J. Zeng Y.-H. Zhu J.-M. Cai Z.-H. Zhou J. (2022). The structure and assembly mechanisms of plastisphere microbial community in natural marine environment. J. Hazard. Mater. 421:126780. doi: 10.1016/j.jhazmat.2021.126780, PMID: 34358974

[ref002] Zhou A. Zhang Y. Xie S. Chen Y. Li J. Wang J.. (2021). Microplastics and their potential effects on the aquaculture systems: a critical review. Reviews in Aquaculture. doi: 10.1111/raq.12496, PMID: 33515883

