# Supplementary material for: The geographical and seasonal effects on the composition of marine microplastic and its microbial communities: The case study of Israel and Portugal
Source: Front Microbiol. 2023 Feb 22;14:1089926. doi: 10.3389/fmicb.2023.1089926 (PMC9992426; doi:10.3389/fmicb.2023.1089926)
Supplement: Supplementary file 1 [file Data_Sheet_1.pdf]

Supporting information 1

Supporting Information (6 pages)

Table S1. Flow meter information for each manta trawl S2

SI-P1 Removal of organic matter from microplastics S2

Figure S1. Sample images of microplastics S2

SI-P2 FTIR to determine polymer composition S3

Table S2. Pairwise comparisons of plastic particles abundances S3

Table S3. Pairwise comparisons of blue plastic particle colors S4

Table S4. Pairwise comparisons of film plastic particles S4

Table S5. Minion sequencing results S5

Figure S2. Alpha diversity S6

Table S6**.** Shared OTUs of plastics S6

Table S1. Flow meter information for each manta trawl.

Location	Date	Time	Duration (min)	Volume (m^3)
Yarkon	03.02	11:29	30	231
Yarkon	03.02	11:55	30	227
Yarkon	03.02	12:23	30	201
Yarkon	13.07	10:25	30	240
Yarkon	13.07	10:52	30	325
Yarkon	13.07	11:26	30	294
Sado	02.03	10:00	30	95.3
Sado	02.03	11:00	29	91.2
Sado	02.03	11:49	32	98.6
Sado	21.07	10:39	29	92.5
Sado	21.07	11:28	30	83.8
Sado	21.07	12:11	31	74.6

SI-P1 Removal of organic matter from microplastics.
10% potassium hydroxide (KOH) (Labchem, 101474-1000) in distilled water was added to the samples at a 3:1 ratio for 48h at room temperature with shaking. After digestion, the samples were filtered through a 12 µm polycarbonate filter (Filterlab® MPC1200047N) using a Millipore vacuum pump (Model wp6111560). Each filter was transferred to a covered and labelled Petri dish for later observation under a stereomicroscope.

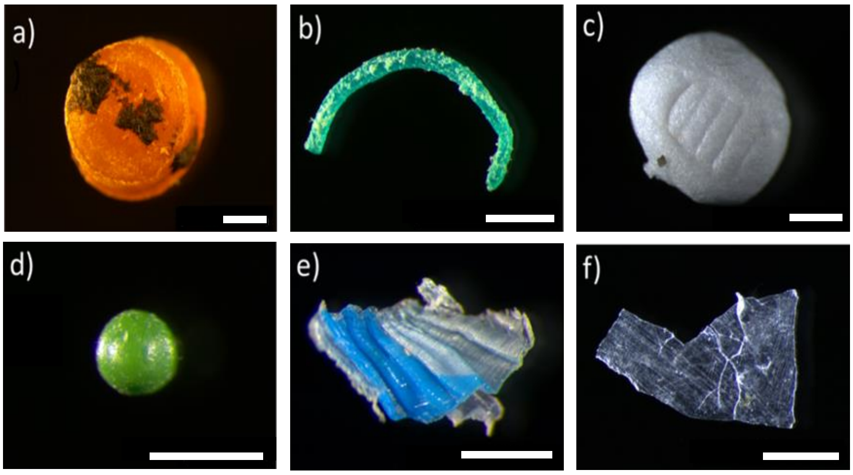

Figure S1. Selected types of microplastics found in the samples from Sado a) pellet, b) filament; c) styrofoam sphere and Yarkon d) microbead, e) fragment f) film.

Table S2. Results of Mann Whitney U-test. Pairwise comparisons of plastic particles abundances. YK: Yarkon; SD: Sado; win: winter; sum: summer. (*) A statistically significant difference (p<0.05).

Pairwise comparisons	p-value
SD sum - YK sum	**0.003***
YK win - YK sum	**0.047***
SD win - YK sum	0.064
SD sum - SD win	0.176
SD sum - YK win	0.508
YK win - SD win	0.659

Table S3. Results of Kruskal-wallis test. Pairwise comparisons of blue plastic particle colors. YK: Yarkon; SD: Sado; win: winter; sum: summer. (*) A statistically significant difference (p<0.05).

Pairwise comparisons	p-value
SD sum - YK sum	0.141
YK win - YK sum	0.299
SD win - YK sum	1.00
SD sum - SD win	**0.006***
SD sum - YK win	1.00
YK win - SD win	**0.043***

Table S4. Results of Kruskal-wallis test. Pairwise comparisons of film plastic particles. YK: Yarkon; SD: Sado; win: winter; sum: summer. (*) A statistically significant difference (p<0.05).

Pairwise comparisons	p-value
SD sum - YK sum	0.108
YK win - YK sum	1.00
SD win - YK sum	0.108
SD sum - SD win	1.00
SD sum - YK win	**0.006***
YK win - SD win	**0.006***

Table S5. Minion sequencing and analysis output of microbial communities on water and plastic using 16S primers. Total reads are all base called reads after nanopore sequencing. Mapped reads are reads after quality control and filtering that are used for taxonomic classification.

Sample		Total reads	Mapped reads	No of OTUs	No of species
Yarkon Winter	Water1	2816	2813	2145	341
	Water2	6308	6269	4323	499
	Water3	13208	13153	9776	1265
	Plastic1	8281	8238	5498	1408
	Plastic2	39329	38694	37116	3842
	Plastic3	13662	13611	10561	1514
Yarkon Summer	Water1	21312	21238	20630	824
	Water2	1384	1380	1306	334
	Water3	19617	19450	18589	1736
	Plastic1	1913	1897	731	294
	Plastic2	14916	14867	7367	1218
	Plastic3	1676	1656	1123	352
Sado Winter	Water1	17079	17066	15103	1430
	Water2	17273	17271	14967	1270
	Water3	22886	22875	20811	1239
	Plastic1	7602	7589	6992	1351
	Plastic2	2110	2105	1981	432
	Plastic3	39329	38694	37116	3842
Sado Summer	Water1	8173	8156	6479	594
	Water2	11589	11568	9011	716
	Water3	8531	8518	6925	557
	Plastic1	3716	3683	2835	732
	Plastic2	6497	6457	4682	1047
	Plastic3	8585	8534	7228	1103

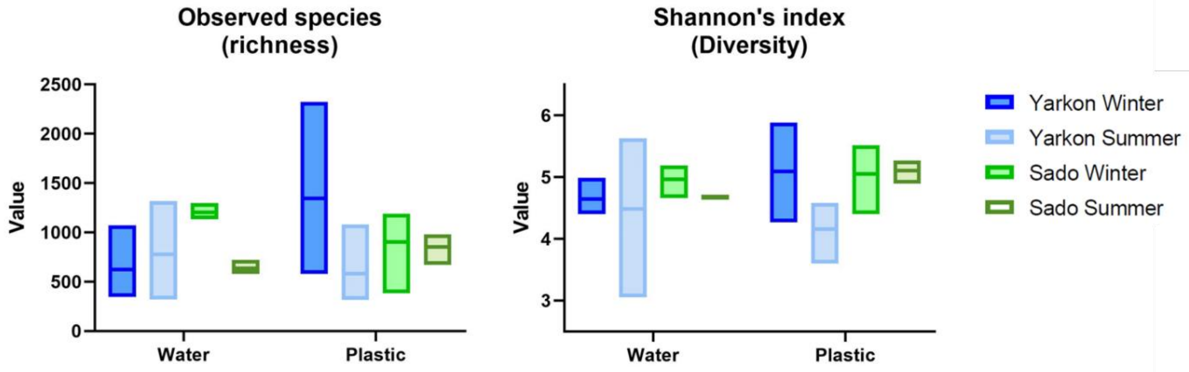

No significance between any comparisons

Figure S2. Alpha diversity of water and plastic communities including richness and diversity. N=3. Box extends from the minimum to the maximum and the line represents the mean. Significance was assessed using two-way ANOVA.

Table S6. Shared bacteria in all plastic samples that were not shared in the water samples (repeats combined).

Class	Family	Species
Alphaproteobacteria	Hyphomonadaceae	*Hellea balneolensis*
Alphaproteobacteria	Rhodobacteraceae	*Aliiroseovarius sediminilitoris*
Alphaproteobacteria	Rhodobacteraceae	*Jannaschia aquimarina*
Alphaproteobacteria	Rhodobacteraceae	*Jannaschia seosinensis*
Alphaproteobacteria	Rhodobacteraceae	*Loktanella hongkongensis*
Alphaproteobacteria	Rhodobacteraceae	*Loktanella ponticola*
Alphaproteobacteria	Rhodobacteraceae	*Maribius pelagius*
Alphaproteobacteria	Rhodobacteraceae	*Oceanicola granulosus*
Alphaproteobacteria	Rhodobacteraceae	*Oceanicola litoreus*
Alphaproteobacteria	Rhodobacteraceae	*Palleronia soli*
Alphaproteobacteria	Rhodobacteraceae	*Pseudooctadecabacter jejudonensis*
Alphaproteobacteria	Rhodobacteraceae	*Pseudoroseicyclus aestuarii*
Alphaproteobacteria	Rhodobacteraceae	*Pseudoruegeria aquimaris*
Alphaproteobacteria	Rhodobacteraceae	*Rhodovulum marinum*

Alphaproteobacteria	Rhodobacteraceae	*Roseivivax isoporae*
Alphaproteobacteria	Rhodobacteraceae	*Roseovarius aestuarii*
Alphaproteobacteria	Rhodobacteraceae	*Sulfitobacter donghicola*
Alphaproteobacteria	Rhodobacteraceae	*Sulfitobacter noctilucicola*
Alphaproteobacteria	Rhodobacteraceae	*Thalassobius aestuarii*
Alphaproteobacteria	Rhodobacteraceae	*Thalassobius litorarius*
Alphaproteobacteria	Sphingomonadaceae	*Erythrobacter citreus*
Alphaproteobacteria	Sphingomonadaceae	*Erythrobacter litoralis*
Alphaproteobacteria	Sphingomonadaceae	*Erythrobacter longus*
Alphaproteobacteria	Sphingomonadaceae	*Erythrobacter nanhaisediminis*
Alphaproteobacteria	Sphingomonadaceae	*Erythrobacter seohaensis*
Alphaproteobacteria	Sphingomonadaceae	*Pacificimonas flava*
Gammaproteobacteria	Alteromonadaceae	*Aliiglaciecola coringensis*
Gammaproteobacteria	Alteromonadaceae	*Alteromonas mediterranea*
Gammaproteobacteria	Pseudomonadaceae	*Pseudomonas putida*

Table S7. Differentially abundant genera and species. FDR cut off < 0.05, negative log2FC indicate higher abundance in Yarkon. Positive log2FC indicate higher abundance in Sado.

**Genus**	log2FC	lfcSE	P. values	FDR
Parvularcula	-6.3007	1.7756	0.000387	0.016133
Acrophormium	-5.9956	1.6488	0.000276	0.01456
Filomicrobium	-4.9255	1.224	5.72E-05	0.005425
Alcanivorax	-4.7088	1.1652	5.32E-05	0.005425
Alteromonas	4.2579	1.1998	0.000387	0.016133
Polaribacter	4.4611	1.3702	0.001131	0.038294
Psychrosphaera	4.6405	1.1999	0.00011	0.008687
Olleya	5.6666	1.6032	0.000408	0.016133
Jannaschia	6.4601	1.757	0.000236	0.01456
Rubrivirga	6.9901	2.0838	0.000795	0.028994
Palleronia	7.3955	2.0233	0.000257	0.01456
Dokdonia	7.792	1.4506	7.81E-08	1.82E-05
Pseudoalteromonas	8.6173	1.6255	1.15E-07	1.82E-05
Fulvimarina	22.01	2.9058	3.60E-14	1.71E-11

**Species**	log2FC	lfcSE	Pvalues	FDR
Alteromonas_genovensis	22.064	2.7549	1.16E-15	7.39E-13
Xenococcaceae_cyanobacterium_CENA315	23.302	3.0037	8.65E-15	2.76E-12
Jannaschia_faecimaris	22.115	2.9319	4.60E-14	9.80E-12
Fulvimarina_pelagi	21.705	2.8946	6.45E-14	9.91E-12
Jannaschia_donghaensis	21.669	2.8991	7.76E-14	9.91E-12
Pseudoalteromonas_sp__BSw20060	7.3844	1.9246	0.000125	0.013277
Pseudoalteromonas_sp__13_15	6.5406	1.8884	0.000533	0.03761
Pseudoalteromonas_sp__ARCTIC_P16	8.3998	2.4441	0.000589	0.03761
uncultured_gamma_proteobacterium_CHAB_IV_34	8.9459	2.4004	0.000194	0.017695
Dokdonia_sp__PRO95	7.0787	1.9312	0.000247	0.019717
Loktanella_sp__S4079	-5.6953	1.6835	0.000717	0.041656
